# Surveillance of Bacterial Meningitis, Ethiopia, 2012–2013 

**DOI:** 10.3201/eid2201.150432

**Published:** 2016-01

**Authors:** Wude Mihret, Tsehaynesh Lema, Yared Merid, Afework Kassu, Workeabeba Abebe, Beyene Moges, Admasu Tenna, Fitsum Woldegebriel, Melaku Yidnekachew, Wondale Mekonnen, Arslan Ahmed, Lawrence Yamuah, Mezgebu Silamsaw, Beyene Petros, Jan Oksnes, Einar Rosenqvist, Samuel Ayele, Abraham Aseffa, Dominique A. Caugant, Gunnstein Norheim

**Affiliations:** Armauer Hansen Research Institute, Addis Ababa, Ethiopia (W. Mihret, T. Lema, M. Yidnekachew, W. Mekonnen, L. Yamuah, S. Ayele, A. Aseffa);; Hawassa University Referral Hospital, Southern Nations and Nationalities Peoples Region, Sodo, Ethiopia (Y. Merid, F. Woldegebriel);; University of Gondar Hospital, Gondar, Ethiopia (A. Kassu, B. Moges, M. Selamsew);; Tikur Anbessa Specialized University Hospital, Addis Ababa, Ethiopia (W. Abebe, A. Tenna);; Norwegian Institute of Public Health, Oslo, Norway (A. Ahmed, J. Oksnes, E. Rosenqvist, D.A. Caugant, G. Norheim);; University of Oslo Faculty of Medicine, Oslo (A. Ahmed, D.A. Caugant);; Addis Ababa University, Addis Ababa (B. Petros)

**Keywords:** bacterial meningitis, Neisseria meningitidis, Streptococcus pneumoniae, meningococci, pneumococci, meningococcal serogroups, bacteria, Ethiopia, real-time PCR

## Abstract

Among 139 patients with suspected bacterial meningitis in Ethiopia, 2012–2013, meningococci (19.4%) and pneumococci (12.9%) were the major disease-causing organisms. Meningococcal serogroups detected were A (n = 11), W (n = 7), C (n = 1), and X (n = 1). Affordable, multivalent meningitis vaccines for the African meningitis belt are urgently needed.

Ethiopia has the second-largest population (≈94 million in 2013) among the meningitis belt countries of sub-Saharan Africa (*1*). However, during 2001–2010, a median of only 1,056 suspected meningitis cases per year (range 5–8,571/year) was reported to the World Health Organization (*2*). The largest meningitis epidemics occurred in 1981 (*3*) and 1989 (*4*), resulting in ≈45,000 and ≈50,000 cases, respectively. Serogroup A meningococci were the major cause of these epidemics, although serogroup C strains were also identified in 1981, 1983–84, and during outbreaks in 2000 and 2003 (*5*). Conjugate vaccines against *Haemophilus influenzae* serotype b, *Streptococcus pneumoniae* (pneumococcal conjugate vaccine [PCV] 10), and *Neisseria meningitidis* serogroup A (MenAfriVac) were introduced in 2007, 2011, and 2013–2015, respectively. Because data permitting assessment of these vaccines are limited, we implemented a surveillance study.

## The Study

**Figure 1:**
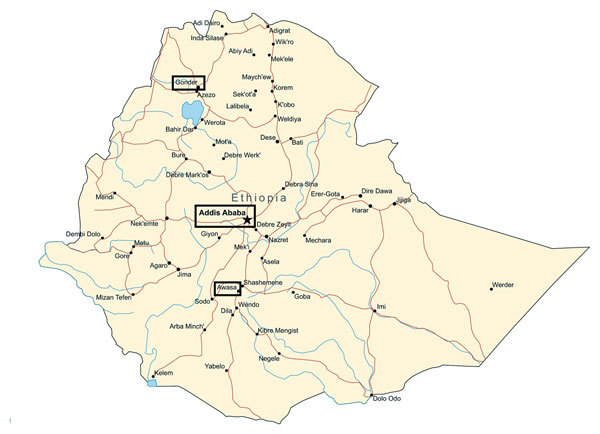
Locations (boxes) of the 3 meningitis surveillance study hospitals in Gondar, Addis Ababa, and Hawassa (also spelled Awasa or Awassa), Ethiopia. Air distances from Addis Ababa to Gondar and Hawassa are ≈420 km and 220 km, respectively. Modified with permission from http://www.MapResources.com.

Patients with symptoms of meningitis admitted to 3 referral teaching university hospitals in Ethiopia (Hawassa Referral Hospital [Southern Nations, Nationalities and Peoples Region], Tikur Anbessa Referral Hospital [Addis Ababa], and Gondar University Hospital [Amhara region]) (Figure 1) during February 2012–June 2013 received a lumbar puncture as part of routine diagnostic procedures. If cerebrospinal fluid (CSF) was turbid, the patient was included in the study. The study was approved by ethical review committees in Norway (Regional Ethics Committee reference 2011/825b) and Ethiopia (National Research Ethics Review Committee reference 3-10/6/5-04).

Demographic and clinical data were recorded in predefined case record forms. CSF samples were inoculated into a transisolate medium vial and sent to Armauer Hansen Research Institute (AHRI) in Addis Ababa for bacteriologic verification by standard procedures (*6*).

Bacterial DNA was extracted by using the QiAmp DNA extraction kit (QIAGEN, Hilden, Germany) according to the manufacturer’s protocol. Conventional multiplex PCR for species identification was performed with primers specific for amplifying the genes *porA* (*N. meningitidis*), *lytA* (*S. pneumoniae*), and *bexA* (*H. influenzae*) for all samples, and capsule genogrouping was carried out for *porA* PCR-positive samples (*7*,*8*). Transisolate samples were also analyzed by multiplex real-time PCR with primers targeting genes *ctrA* (*N. meningitidis*), *ompP* (*H. influenzae*), and *lytA* (*S. pneumoniae*). Real-time PCR reactions (3 parallel) were run on an ABI 7500 fast real-time PCR system (Applied Biosystems, Foster City, CA, USA); a cycle threshold (C_t_) value of <40 indicated a positive result. If C_t_ was >35, the sample was retested. Samples positive for the *ctr*A gene were subjected to capsule genogrouping by singleplex real-time PCR for verification of *N. meningitidis* serogroups A, B, C, Y, W, and X (*9*). CSF samples positive for *N. meningitidis* were further tested in a nested *porA* PCR, followed by sequencing the *porA* gene to identify the 2 variable region peptide loops of the PorA protein (*5*). Multilocus sequence typing and ferric enterobacin transport genotyping were performed (*6*). 

During the surveillance period, 139 patients met criteria for suspected bacterial meningitis and were included in our analysis (Table 1). Of these, 92 patients (66.2%) were admitted in Gondar, 27 in Hawassa (19.4%), and 20 in Addis Ababa (14.4%). Culturing performed at AHRI identified a pathogen in 15 (10.8%) of the 139 patients: *N. meningitidis* (n = 4), *S. pneumoniae* (n = 9), and *H. influenzae* (n = 1). Conventional multiplex PCR performed at AHRI identified DNA from the same 3 pathogens in 18 (12.9%) CSF samples: *N. meningitidis* (n = 7), *S. pneumoniae* (n = 10), and *H. influenzae* (n = 1). By multiplex real-time PCR of the same CSF samples, etiologic agent could be verified in 46 (33.1%) samples: *N. meningitidis* (n = 27; 19.4%), *S. pneumoniae* (n = 18; 12.9%), and *H. influenzae* (n = 1; 0.7%). For the remaining 93 patients, an etiologic agent for the meningitis episode was not determined. 

**Table 1:** Age and sex distribution of 139 patients with suspected bacterial meningitis and breakdown of identified pathogen types, Ethiopia, 2012–2013

Patient characteristic	Total no. (%) cases	No. (%) *Neisseria meningitidis* infections, n = 28	No. (%) *Streptococcus pneumoniae* infections, n = 18
Age, y			
≤4	48 (34.5)	9 (33.3)	6 (33.3)
5–12	26 (18.7)	11 (40.7)	4 (22.2)
13–19	13 (9.4)	4 (14.8)	1 (5.6)
20–29	20 (14.4)	2 (7.4)	4 (22.2)
30–39	12 (8.6)	1 (3.7)	0
≥40	20 (14.4)	0	3 (16.7)
Sex			
M	83 (59.7)	17 (63.0)	10 (55.6)
F	56 (40.3)	10 (37.0)	8 (44.4)

The proportion of CSF samples with etiologic agent identified by real-time PCR varied between sites, peaking in Hawassa with 19 (70.4%) of 27 samples, followed by Addis Ababa with 7 (35.0%) of 20 samples and Gondar with 20 (21.7%) of 92 samples (Table 2). Of 27 CSF samples positive for *N. meningitidis*, genogroup could be determined for 20 (Table 2). For the remaining 7 samples, genogroup could not be determined by real-time PCR because DNA concentration was low. 

**Table 2:** Organisms detected by real-time PCR in CSF samples from 139 meningitis patients, by location, Ethiopia, 2012–2013*

Location	*Neisseria meningitidis*† serogroup					*Streptococcus pneumoniae*‡	*Haemophilus influenzae*§
	A	C	W	X	NG		
Gondar	0	1	6	0	3	10	0
Hawassa	9	0	1	1	2	5	1
Addis Ababa	2	0	0	0	2	3	0
Total	11	1	7	1	7	18	1

*CSF, cerebrospinal fluid; NG; nongroupable. †n = 27. ‡n = 18. §n = 1.

**Figure 2:**
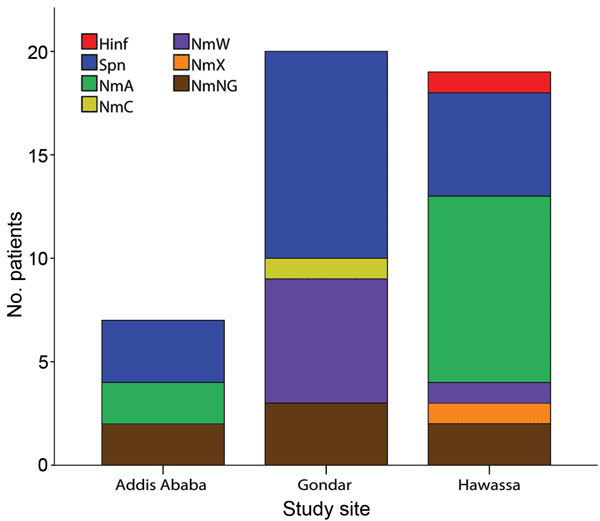
Distribution of causative organisms among 139 patients with clinical symptoms of bacterial meningitis in Ethiopia, 2012–2013, as verified by DNA from either meningococci (*Neisseria meningitidis,* serogroups A, NmA; C; NmC; X, NmX; ; W, NmW; and NG, not serogrouped as A, C, Y, W, or X), *Streptococcus pneumoniae* (Spn), or *Haemophilus influenzae* (Hinf) in cerebrospinal fluid.

Serogroup distribution differed substantially by geographic region: W dominated in Gondar, A in Hawassa (Figure 2). One case of meningococcal disease caused by serogroup C and 1 caused by serogroup X meningococci were identified in Gondar and Hawassa, respectively. *PorA* genosubtyping results were available for 19 of the 27 CSF samples containing *N. meningitidis* DNA; both PorA variable regions were indicated for 15 samples. Samples from Hawassa were P1.20,9 (n = 7), P1.5–11,10–1 (n = 1), P1.5,_ (n = 1) and P1._,9 (n = 1), whereas those from Gondar were P1.5,2 (n = 7), P1._4 (n = 1) and P1._,2 (n = 1). Genotyping of the 4 meningococcal strains isolated showed that 1 was serogroup W, P1.5,2:F1–1:ST11, whereas 3 were serogroup A, P1.20,9:F3–1:ST7. The 3 pneumococcal isolates that were recovered for multilocus sequence typing were sequence types (ST) 8875 (n = 2) and ST289 (n = 1), all from Gondar. 

The case-fatality rate (CFR) for meningococcal patients was 11.1% (3/27), whereas that for pneumococcal patients was 16.7% (3/18). Among the total 20 fatal bacterial meningitis cases, 8 were reported from Hawassa; 2 were caused by pneumococci, 3 by meningococci (1 serogroup A, 1 nongroupable, and 1 W), and 1 by *H. influenzae*. Of the 2 fatal cases from Addis Ababa, 1 was caused by *S. pneumoniae.* No samples from fatal cases from Gondar were positive by real-time PCR. The proportion of meningococcal case-patients with serogroup A infection in the MenAfriVac target group (1–29 years of age ) was 31.0% (9/29).

These case-based demographic data and laboratory-verified analyses of CSF samples from 139 bacterial meningitis patients in 3 hospitals in Ethiopia indicate baseline data before MenAfriVac vaccination. The dominance of serogroups W and A among the cases of known etiology in Gondar and Hawassa, respectively, suggests geographic variation in meningoccocal serogroup distribution in Ethiopia (Figure 2). The presence of serogroup W and X in Ethiopia is in line with trends in the rest of the meningitis belt (*10*–*12*) and may diminish the effects of the monovalent serogroup A conjugate vaccine on overall meningococcal disease incidence. Molecular typing showed that serogroup A meningococci isolated in Ethiopia in 2012–2013 were the same ST (ST7) as those causing the 2002–2003 outbreaks; both expressed PorA P1.20,9 (*4*,*5*). The serogroup W isolates were ST11 with PorA P1.5,2, the same found among outbreak strains in other meningitis belt countries (*11*–*13*). 

Variation between sites and the overall low rates of etiologic agent identification may be explained by differences in interpreting meningococcal symptoms and CSF turbidity, as well as delay in transporting samples to the laboratory. CFR among meningococcal disease patients (11.1%) was comparable with that observed in other meningitis belt countries, whereas CFR among pneumococcal disease patients (16.7%) was lower than typically observed (≈50%) (*14*).

## Conclusions

This study highlights the need for reinforcement of case-based, laboratory confirmed surveillance of bacterial meningitis in Ethiopia to enable mapping of distribution of causative organisms across the country and determine the potential effects of existing vaccines. The high proportion of serogroup W meningococci observed in northern Ethiopia is cause for concern, as is the presence of serogroup X. Recent outbreaks of meningitis caused by serogroup W in Burkina Faso and C in Nigeria (*15*) have been met with reactive vaccination campaigns with polysaccharide vaccines in areas where MenAfriVac has been implemented. This situation is suboptimal and calls for fast-tracking the development of affordable, multivalent conjugate vaccines against serogroups A, C, Y, W, and X meningococci (*10*).
